# Rapid PROTAC discovery, design and testing by coupling crystallography and biology

**DOI:** 10.1063/4.0000807

**Published:** 2025-10-27

**Authors:** Debanu Das, Thomas Pesnot

**Affiliations:** 1 Accelero Biostructures, San Carlos, CA, USA; 2 XPose Therapeutics, San Carlos, CA, USA; 3 Concept Life Sciences, Chapel-en-le-Frith, High Peak, United Kingdom

## Abstract

Proteolysis targeting chimeras (PROTACs) in targeted protein degradation (TPD) is an exciting emerging therapeutic modality in drug discovery. PROTACs include three components: a target-specific warhead for the protein of interest; a chemical linker; and an E3 ubiquitin ligase ligand connected to the target warhead-linker combination. The resulting induced proximity results in an E3 ubiquitin ligase like Cereblon or pVHL, ubiquitinating the protein of interest for selective proteasomal degradation resulting in the depletion of cellular levels of the target protein. There are two key steps in developing a PROTAC against a target protein, which are identifying an appropriate small molecule warhead for the target and introducing an appropriate linker for the E3 Ligase-ligand portion. We have developed methods that can vastly simplify and speed up the discovery and development of PROTACs for TPD, providing a range of options and opportunities for new drug discovery research.

Our approach starts with our hit generation by our proprietary high-throughput protein X-ray crystallography-based library screening of hundreds of compounds in a few days as a primary screen. Hits are then evolved rapidly into lead warheads in a few weeks. The prioritized warheads are coupled to our proprietary E3 ligase library in a parallel fashion to afford hundreds of putative degraders, which are tested in bioassays without intermediate purification. This high-throughput Direct-to-Biology (D2B) approach provides means to generate and characterize a large array of putative PROTACs in a matter of days, hence accelerating the PROTAC discovery process by several orders magnitude compared to traditional methodologies.

We will discuss this approach in the context of drug discovery on three DNA Damage Response proteins APE1, LINE-1 EN, POLH and FEN1.

